# Long-term clinical outcomes of Zika-associated Guillain-Barré syndrome

**DOI:** 10.1038/s41426-018-0151-9

**Published:** 2018-08-22

**Authors:** Aileen Y. Chang, Rebecca Lynch, Karen Martins, Liliana Encinales, Andrés Á. Cadena Bonfanti, Nelly Pacheco, St. Patrick Reid, Osvaldo E. Lara Sarabia, Henry J. González Torres, Stella Mejia Castillo, Onaldo Barrios Taborda, Magda Alarcon Gomez, Brenda Guerra Duran, Victor Martinez Giraldo, Angélica Benitez Ospino, Alexandra Porras, Alejandro R. Mendoza, Grace Mantus, Guangzhao Li, Jin Peng, Priyanka Kamalapathy, Lil G. Avendaño Echavez, Kimberly A. Dowd, Monica Rengifo-Pardo, Pedro Pablo Barraza, Dennys Jiménez Hernàndez, Andrés González Coba, Katya De La Hoz Mendoza, Jeffrey M. Bethony, Gary L. Simon

**Affiliations:** 1 The George Washington University, Washington, DC 20037 USA; 2 United States Army Medical Research Institute for Infectious Diseases, Fort Detrick, MD USA; 3 Universidad Simón Bolívar, Barranquilla, Atlántico Colombia; 4 Clinica de La Costa Ltda, Baranquilla, Atlántico Colombia; 5 Allied Research Society LLC, Baranquilla, Atlántico Colombia; 6 University of Nebraska Medical Center, Omaha, NE USA 68182; 7 Universidad El Bosque, Bogotá, Colombia; 8 National Institutes of Health, Bethesda, MD USA 20892

Zika virus infection has been associated with the development of a spectrum of neurologic disease including Guillain–Barré syndrome (GBS)^1^. GBS is an autoimmune disorder of the peripheral nervous system often triggered by a preceding infection. The mechanism of Zika-associated GBS (Z-GBS) and the long-term clinical course is unknown. The purpose of this study was to describe the 2-year clinical course of Z-GBS in order to provide further insights into disease pathogenesis and prognosis.

Clinical and demographic characteristics of the 18 patients infected during the Colombian Zika epidemic^1^ with serologically diagnosed Zika infection with GBS are reported in Supplemental Table 1. The Z-GBS cases were mestizo adults, median age 50, and 63% male. Dengue-neutralizing antibodies were found in 100% of patients. Sixty-eight percent of the patients reported Zika viral symptoms with the most common symptoms being arthralgia (72%), myalgia (72%), fever (67%), and rash (67%). The median time from Zika to neurologic symptom onset was 7 days (interquartile range (IQR) 5–21).

The clinical course of neurologic disease among Z-GBS cases demonstrated the rapid decline from onset of neurologic symptoms to disease nadir in median 5 days and improvement at a median 17 days (Supplemental Table 2). Patients suffered neurologic symptoms for a median total of 27 days. The most common neurologic symptoms were muscular weakness (100%), primarily lower extremity weakness (79%), inability to walk (89%), paresthesias (84%), sensory deficit (84%), and diminished reflexes (80%).

Cerebrospinal fluid analysis revealed elevated protein (91%), less than 4 white blood cells (100%), and negative bacterial culture (100%). Electrodiagnostic study results showed acute inflammatory demyelinating polyneuropathy (AIDP) (43%), acute motor axonal neuropathy (AMAN) (13%), sensory motor demyelinating polyneuropathy with axonal involvement (38%), and one patient with Miller Fisher variant. Patients were treated with intravenous immunoglobulin (89%) and/or plasmapheresis (24%). Cases were hospitalized a median of 20 days and spent a median of 6 days in the intensive care unit. None of the patients died.

Hughes disability score^2^ after 1 year (*n* = 15) demonstrated that 60% of patients were healthy, 20% had minor symptoms but were capable of running, 13% were able to walk 5 m across an open space with support, and one patient was confined to a wheelchair (Table 1). After 2 years (*n* = 13), 38% of patients were healthy, 15% had minor symptoms but were capable of running, 31% were able to walk 5 m across an open space with support, and one patient was still confined to a wheelchair. The Clinical Health Assessment Questionnaire-II (CLIN-HAQII) Score is a composite score of disability ranging from 0 (minimal loss of function) to 3 (completely disabled). Z-GBS cases demonstrated overall minimal loss of function (Median CLINA-HAQII score: [Year 1: 0, Year 2: 0.09]). Patients reported minimal pain on a scale from 0 (no pain) to 100 (maximum pain) [Median pain score: [Year 1: 5, Year 2: 40] and general wellbeing scale from 0 (“I feel well”) to 100 (“I feel very bad”) [Median wellbeing score: [Year 1: 4, Year 2: 21]. Only 20% of patients were depressed 1 year after their neurologic disease, but this increased 62% after 2 years. Paired analysis of quantitative variables indicated no significant differences in CLIN-HAQII, pain, or well-being scores from Year 1 to Year 2.

**Table 1:** Disability characteristics of serologically diagnosed Zika Guillain–Barré syndrome (Z-GBS) cases with Brighton Criteria level 1 or 2 at 1 (*n* = 15) and 2 (*n* = 13) years follow-up post-GBS

**Characteristics**	**1 Year follow-up**	**2 Year follow-up**
**Hughes Disability Score** ^2^ **, n/d (%)**		
Healthy	9/15 (60%)	5/13 (38%)
Minor symptoms but capable of running	3/15 (20%)	2/13 (15%)
Able to walk 5 m across an open space with support	2/15 (13%)	4/13 (31%)
Confined to bed or chair bound	1/15 (7%)	1/13 (8%)
HAQ-II Score^12^, median (IQR)	0 (0–0.08)	0.09 (0–0.55)
Use of wheelchair*,* n/d (%)	1/15 (7%)	1/13 (7%)
Pain score in last week, median (IQR) from 0 none to 100 maximum pain	5 (0–30)	40 (0–60)
General wellbeing score, median (IQR) from 0 “I feel well” to 100 “I feel very bad”	4 (0–20)	21 (0–50)
**Mental health, n/d (%) in the last month**		
Frequently enjoying things	14/15 (93%)	12/13 (92%)
Frequently feel calm	11/15 (73%)	11/13 (85%)
Frequently feel nervous	2/15 (13%)	9/13 (69%)
Frequently feel depressed	3/15 (20%)	8/13 (62%)
Frequently feel nothing is going well	3/15 (20%)	5/13 (38%)

^a^Clinical Health Assessment Questionnaire 2 (CLIN-HAQ2) is a composite score between 0 (minimal loss of function) and 3 (completely disabled)^13^

In this report to describe the clinical course of Z-GBS with 2-year follow-up, our primary findings were that the majority of patients had a full recovery but suffered neurologic symptoms for a significant time period (median of 27 days (IQR 12–46)). After 1 year, the Hughes disability score demonstrated that only 60% of patients were healthy with 40% with some disability. This finding is comparable to classic GBS with 32–65% experiencing some disability at 1-year post-GBS^3^. In contrast, in a cohort of seven patients with GBS post-Varicella-zoster virus infection with demyelinating disease, all of the patients were able to run at 1-year follow-up^4^. Two years after GBS, disability, pain, and general wellbeing scores were not significantly different indicating persistent disabilities in a small subset of patients.

Multiple additional observations can be drawn from this data that may shed light on Z-GBS pathophysiology. First, the time course from Zika infection to GBS onset of median 7 days (IQR 5–21) is consistent with a direct viral or antibody-mediated pathophysiology and in accord with other studies in which a prior viral illness occurred a median of 5–10 days before GBS onset^1,5–7^.

Molecular mimicry is a potential mechanism whereby anti-ZIKV antibodies target peripheral nerve glycolipids. The time course from ZIKV infection to GBS symptoms could be consistent with anti-ZIKV antibody production or boosting of prior anti-DENV antibodies in response to ZIKV infection. Interestingly, all of the Z-GBS patients in this cohort also demonstrated prior DENV infection. We have previously shown that Z-GBS is associated with higher anti-ZIKV and anti-DENV-neutralizing antibodies compared to ZIKV patients without GBS suggesting a potential role for antibodies in the development of Z-GBS (pending publication).

The electrodiagnostic results shed further light on the pathophysiology. Our results showed nerve conduction studies consistent with AIDP and AMAN. This is consistent with other case series of Zika-GBS patients demonstrating the majority of patients with AIDP with a few AMAN-type GBS^1,6,7^. Interestingly we also found a sensory component with approximately one-third of patients with sensory motor demyelinating polyneuropathy with axonal involvement (35%) and sensory deficits and paresthesias in 84% of patients, consistent with other studies demonstrating 74–90% of patients with paresthesias^1,5–7^. This demonstrates the growing evidence of sensory involvement in GBS^8,9^.

The limitations of this analysis include retrospective identification of patients such that documentation available for some patients to confirm Brighton criteria level 1 or 2 was limited resulting in exclusion of 13 patients. Since real-time polymerase chain reaction (PCR) was not possible because patients were out of the window for PCR detection of viral infection by the time of blood draw, neutralizing antibodies were used to confirm Zika infection which is the most specific serologic test available to date. While the patients had symptoms and serology consistent with ZIKV infection during a local ZIKV epidemic, other triggers of GBS cannot be ruled out. Five patients had diarrhea which can be found in PCR confirmed Z-GBS^1^ but also *Campylobacter jejuni* infection which is a well-known cause of GBS. Unfortunately, acute sera and stool samples were not available for *Campylobacter jejuni* diagnosis; however, the clinical symptoms of these patients were more consistent with ZIKV infection including conjunctivitis (60%) and eye pain (80%) which are not common symptoms of *Campylobacter jejuni*. Finally, GBS associated with Zika infection does not indicate that the GBS is caused by ZIKV infection. Further research in a controlled setting is needed to evaluate causative mechanisms.

## Methods

## Ethics statement

This study (IRB#121611, Trans #28283) was approved by the ethics committee of the Clinica de La Costa Ltda., the George Washington University Committee on Human Research and exemption was provided for analysis of de-identified samples by the Office of Human Use and Ethics from the Department of Army, United States Army Medical Research Institute of Infectious Diseases. All data were collected into REDCap secure database, a Health Insurance Portability and Accountability Act of 1996 (HIPAA) compliant, password protected online database.

## Participants and setting

Forty-two adult patients with a clinical diagnosis of GBS were referred from several departments in northern Colombia during a 2015–2016 Zika epidemic (Supplemental Figure 1). Thirty-two were serologically diagnosed via detection of Zika neutralizing antibodies. Nineteen met Brighton criteria^10^ level 1 or 2 diagnostic certainty of GBS and were included in this analysis. Follow-up evaluation of patients 1-year (*n* = 15) and 2-year (*n* = 13) post-GBS is reported.

## Serologic ZIKV infection determination

Serologic confirmation of Zika infection was performed using the reporter virus particle (RVP) neutralization assay^11^. To evaluate the extent of ZIKV/DENV cross neutralization in our assay, ZIKV neutralization titers (90% inhibitory dilutions (ID90)) among 11 Latin American DENV cases diagnosed prior to ZIKV circulation in the Western hemisphere were used as controls. Median ZIKV neutralization using the RVP assay among these ZIKV naïve DENV cases was 0 with an interquartile range (0–14) (Supplemental Table 3). Therefore, there was minimal cross reactivity between DENV and ZIKV in this RVP assay.

ZIKV infection was defined as a ZIKV H/PF ID90 > 50. The ID90 were defined as the reciprocal sera dilution resulting in a 90% reduction in infectivity. Dengue II 16681 RVP was also performed with positivity also defined as an ID90 >50.

## Statistical analysis

Descriptive statistics including median and interquartile ranges for quantitative variables are shown. SAS (version 9.3, Cary, NC) was used for data analysis.

## Electronic supplementary material

Long-term clinical outcomes of Zika-associated Guillain-Barré syndrome

## Data Availability

All data are available to any interested parties by emailing the corresponding author.
