# Supplementary material for: Long-term clinical outcomes of Zika-associated Guillain-Barré syndrome
Source: Emerg Microbes Infect. 2018 Aug 22;7:148. doi: 10.1038/s41426-018-0151-9 (PMC6104068; doi:10.1038/s41426-018-0151-9)
Supplement: Supplementary file 1 — Long-term clinical outcomes of Zika-associated Guillain-Barré syndrome [file 41426_2018_151_MOESM1_ESM.doc]

**Supplemental Figure 1. Study flow diagram.** During the Colombian Zika virus (ZIKV) epidemic, 2015- 2016, 42 cases of Guillain-Barré syndrome (GBS) were referred for this study. 32 GBS cases were serologically diagnosed for ZIKV exposure via detection of ZIKV neutralizing antibodies. 19 cases serologically diagnosed ZIKV GBS cases had Brighton criteria level 1 or 2 certainty of GBS diagnosis. The clinical information of these patients was included in analysis with clinical follow-up data available from patients one (n=15) and two years (n=13) post-GBS.

**Guillain-Barré syndrome (GBS) Cases referred during Zika epidemic**

**(n = 42)**

**Excluded (n = 10)**

**No serologic evidence of Zika infection**

**Serologically diagnosed Zika GBS cases**

**(n = 32)**

**Excluded (n = 13)**

**Brighton criteria >level 2**

**ANALYZED CASES**

**Serologically diagnosed Zika GBS cases with Brighton criteria level 1 or 2**

**(n = 19)**

**Lost to ONE-YEAR follow-up (n=4)**

**ONE-YEAR POST-GBS**

**Serologically diagnosed Zika GBS cases with Brighton criteria level 1 or 2**

**followed at median –months post GBS**

**(n=15)**

**(n = 11)**

**Lost to TWO-YEAR follow-up (n=2)**

**TWO YEARS POST-GBS**

**Serologically diagnosed Zika GBS cases with Brighton criteria level 1 or 2**

**followed at median –months post GBS**

**(n=13)**

**(n = 11)**

**Supplemental Table 1**. Clinical and Demographic Characteristics of 19 Patients with Serologically diagnosed Zika and Guillain–Barré Syndrome with Brighton Criteria level 1 or 2.

| **Characteristic** | **Value**  N=19 |
| --- | --- |
| **Median Age** –*years (IQR)* | 50 (39-58) |
| **Male gender** –*n/d (%)* | 12/19 (63%) |
| **Mestizo Ethnicity**- *n/d (%)* | 15/15 (100%) |
| **At least some secondary school**- *n/d (%)* | 11/15 (73%) |
| **Comorbidities**- *n/d (%)* | 9/19 (47%) |
| Pregnancy | 0/19 (0%) |
| Obesity | 1/19 (5%) |
| Diabetes | 1/15 (7%) |
| Smoking | 2/19 (11%) |
| Hypertension | 5/19 (26%) |
| Heart Disease | 2/19 (11%) |
| **Viral symptoms** –*n/d (%)* | 13/19 (68%) |
| Arthralgia | 13/18 (72%) |
| Myalgia | 13/18 (72%) |
| Fever | 12/18 (67%) |
| Rash | 12/18 (67%) |
| Headache | 9/15 (60%) |
| Eye pain | 9/18 (50%) |
| Conjunctivitis | 6/18 (33%) |
| Diarrhea | 5/18 (28%) |
| Vomiting | 2/15 (13%) |
| **Median time from onset of ZIKV infection symptoms to onset of GBS**-  *Days (IQR)* | 7 (5-20) |
| **Neurologic diagnosis** – n (%) |  |
| Guillain–Barré syndrome Brighton  criteria level 1 | 12/19 (63%) |
| Guillain–Barré syndrome Brighton  criteria level 2 | 7/19 (37%) |
| Exposure to dengue, neutralizing antibodies 90% inhibitory dilution to DENV >50 | 19/19 (100%) |

**Supplemental Table 2**. Clinical Neurologic Characteristics of 19 Patients with Serologically diagnosed Zika and Guillain–Barré Syndrome with Brighton Criteria level 1 or 2.

| Finding | Value |
| --- | --- |
| **Time course**- *median days (IQR)* |  |
| Days from onset of neurologic symptoms to nadir n=19 | 5 (3-8) |
| Days from onset of neurologic symptoms to beginning of improvement n=18 | 17 (12-27) |
| **Neurologic Symptoms** *– n/d (%)* |  |
| Muscle weakness | 19/19 (100%) |
| Upper extremity paralysis | 13/19 (68%) |
| Lower extremity weakness | 15/19 (79%) |
| Inability to walk | 16/18 (89%) |
| Diminished reflexes | 12/15 (80%) |
| Facial paralysis | 5/19 (26%) |
| Double vision | 2/15 (13%) |
| Vision loss | 2/15 (13%) |
| Muscle rigidity | 6/15 (40%) |
| Sensory loss | 16/19 (84%) |
| Difficulty swallowing | 8/15 (53%) |
| Paresthesias | 16/19 (84%) |
| Difficulty breathing | 13/19 (68%) |
| Neuropathic pain | 7/15 (47%) |
| Ataxia | 13/15 (87%) |
| Vertigo | 4/15 (27%) |
| Convulsions | 0/15 (0%) |
| Cognitive impairment | 0/15 (0%) |
| Neuropsychiatric impairment | 2/15 (13%) |
| **Cerebrospinal fluid results** |  |
| Elevated protein | 10/11 (91%) |
| Less than 4 white blood cells | 13/13 (100%) |
| Negative bacterial culture | 11/11 (100%) |
| **Electrodiagnostic study results** |  |
| Acute inflammatory demyelinating polyneuropathy (AIDP) | 7/16 (43%) |
| Acute Motor Axonal Neuropathy (AMAN) | 2/16 (13%) |
| Sensory motor demyelinating polyneuropathy with axonal involvement | 6/16 (38%) |
| Miller Fisher variant | 1/16 (6%) |
| **Treatment** *– n/d (%)* |  |
| Intravenous immunoglobulin | 17/19 (89%) |
| Plasmapheresis | 4/17 (24%) |
| **Health Care Utilization** – *median days (IQR)* |  |
| Sick Days | 27 (12-46) |
| Days Hospitalized | 20 (17-41) |
| Days in the Intensive Care Unit | 6 (0-15) |

**Supplemental Table 3**. Reporter Virus Particle Neutralization Assay Titers

| Patient Group | Titer- *median days (IQR)* | |
| --- | --- | --- |
|  | DENV-2 | ZIKV H/PF |
| Guillain-Barré cases after ZIKV infection | 652 (276-1534) | 206 (140-491) |
| Dengue cases (ZIKV naïve) | 1310 (442-4231) | 0 (0-14) |
